# Expression Profiling of Circulating MicroRNAs in Canine Myxomatous Mitral Valve Disease

**DOI:** 10.3390/ijms160614098

**Published:** 2015-06-19

**Authors:** Qinghong Li, Lisa M. Freeman, John E. Rush, Dorothy P. Laflamme

**Affiliations:** 1 Nestle Purina Research, Saint Louis, MO 63164, USA; E-Mail: dorothy.laflamme@rd.nestle.com; 2 Cummings School of Veterinary Medicine, Tufts University, North Grafton, MA 01536, USA; E-Mails: Lisa.Freeman@tufts.edu (L.M.F.); john.rush@tufts.edu (J.E.R.)

**Keywords:** microRNA, dog, congestive heart failure, biomarker, myxomatous mitral valve disease, RT-qPCR

## Abstract

MicroRNAs (miRNAs) are small non-coding RNAs that have shown promise as noninvasive biomarkers in cardiac disease. This study was undertaken to investigate the miRNA expression profile in dogs with myxomatous mitral valve disease (MMVD). 277 miRNAs were quantified using RT-qPCR from six normal dogs (American College of Veterinary Internal Medicine Stage A), six dogs with MMVD mild to moderate cardiac enlargement (ACVIM Stage B1/B2) and six dogs with MMVD and congestive heart failure (ACVIM Stage C/D). Eleven miRNAs were differentially expressed (False Discovery Rate < 0.05). Dogs in Stage B1/B2 or C/D had four upregulated miRNAs, including three *cfa-let-7*/*cfa-miR-98* family members, while seven others were downregulated, compared to Stage A. Expression of six of the 11 miRNAs also were significantly different between dogs in Stage C/D and those in Stage B1/B2. The expression changes were greater as disease severity increased. These miRNAs may be candidates for novel biomarkers and may provide insights into genetic regulatory pathways in canine MMVD.

## 1. Introduction

MicroRNAs (miRNAs) are small (~22 nucleotide) single-stranded non-coding RNA molecules that negatively regulate gene expression by promoting degradation of mRNA transcripts or inhibition of protein translation [1]. It has been estimated that over 60% of human protein-coding genes are regulated by miRNAs [2]. According to the miRBase (www.mirbase.org) [3], 2588 and 1915 mature miRNAs have been identified in humans and mice respectively, but to date only 453 have been identified in dogs.

Myxomatous mitral valve disease (MMVD) affects approximately 9% of all dogs, increasing with age such that the overall cumulative incidence is greater than 40% [4,5]. Although the echocardiographic, pathological, and histological changes have been well documented, the molecular changes contributing to MMVD remain unclear. Although serum concentrations of natriuretic peptides increase in dogs with MMVD and CHF [6,7], additional biomarkers may enhance our knowledge about molecular changes and mechanisms in this common disease.

MiRNAs are emerging as potential biomarkers because of their specific expression in many diseases [2], their remarkable stability [8], and the fact that they are found in most tissue types and body fluids [8,9]. Gene expression studies have shown that miRNAs are differentially expressed in heart disease [10] and there is considerable evidence for an important role of miRNAs in cardiac remodeling and congestive heart failure (CHF) [11,12,13,14]. Gerling *et al.* documented a correlation in gene expression profiles between heart tissue and peripheral blood in a rat model of cardiac failure. Their findings supported a correlation between heart and blood transcriptomics [15]. Liew *et al.* showed that human blood expresses tissue-specific transcripts compared to various tissues, including cardiac [16]. Even though *miR-499* is expressed almost exclusively in heart tissue, plasma *miR-499* concentrations were significantly elevated in human patients with myocardial infarction compared with other groups of patients [17]. These findings suggest that circulating biomarkers can serve as appropriate surrogates for the heart tissues in the study of cardiac disease [18].

To our best knowledge, there are four reports on the expression profiling of miRNAs in canine cardiac disease and CHF to date. Only two investigated circulating miRNAs. Steudemann *et al.* evaluated circulating miRNA expression profiles in the serum from Doberman pinschers with and without dilated cardiomyopathy, but found no significant differences [19]. Hulanicka *et al.* analyzed the expression of nine preselected miRNAs in the plasma of Dachshunds with MMVD and identified two significantly downregulated miRNAs: *cfa-miR-30b* in Stage B and *cfa-miR-133b* in Stage C [20]. A third study examined time-dependent expression changes in heart tissues in an experimental canine model of CHF [21]. Despite the generally assumed roles in human ventricular remodeling, *cfa-miR-1*, *cfa-miR-133*, and *cfa-miR-208* expressions were not altered in left ventricle (LV) cardiomyocytes from dogs. In a most recent study, Zhang *et al.* reported 16 miRNAs differentially expressed between the control dogs and dogs with atrial fibrillation and proposed a novel role of *cfa-miR-206* in canine atrial fibrillation [22]. Despite this progress, more research is urgently needed to advance our understanding of the role of miRNAs in canine cardiac disease and CHF.

## 2. Results and Discussion

### 2.1. Differentially Expressed miRNAs

**Figure 1:**
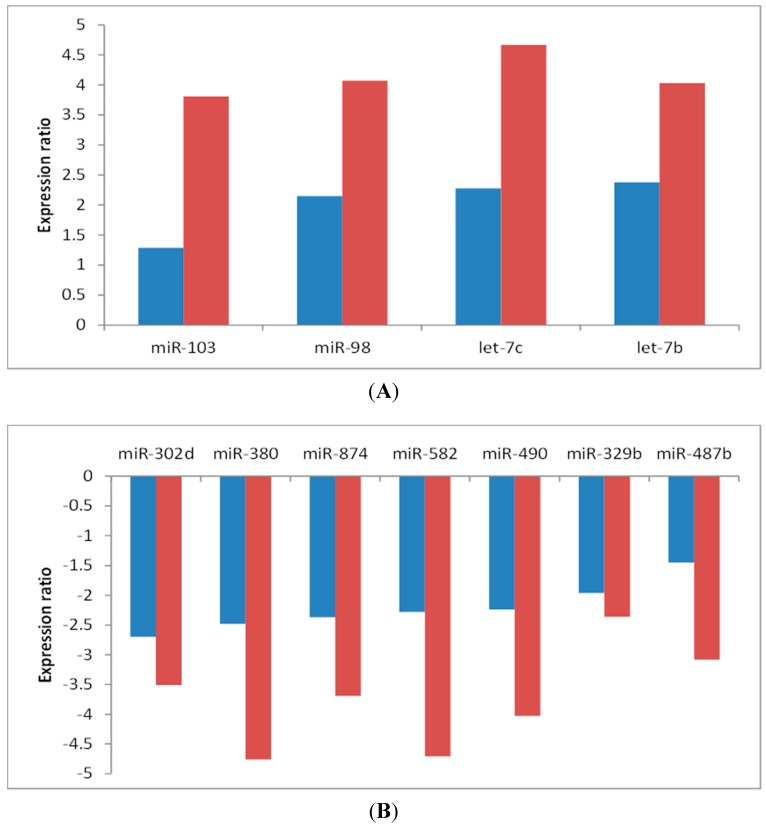
Expression ratios of differentially expressed microRNAs in the serum of dogs with myxomatous mitral valve disease (MMVD). Groups of six dogs each included normal dogs at risk of heart disease (Stage A), asymptomatic dogs with MMVD and mild to moderate cardiac enlargement (Stage B1/B2) and dogs with MMVD and congestive heart failure requiring multiple cardiac medications (Stage C/D). (**A**) shows miRNAs upregulated in dogs with stage B1/B2 or stage C/D compared to stage A; and (**B**) shows miRNAs downregulated in dogs with stage B1/B2 or stage C/D compared to stage A. Blue bars indicate the changes in stage B1/B2 and red bars indicate the changes in stage C/D, compared to stage A.

Of 277 miRNAs evaluated, Analysis of Variance (ANOVA) analysis identified 11 miRNAs with False Discovery Rate (*FDR*) <0.05 (Table 1). Among those, seven miRNAs (*cfa-miR-302d*, *cfa-miR-380*, *cfa-miR-874*, *cfa-miR-582*, *cfa-miR-490*, *cfa-miR-329b*, and *cfa-miR-487b*) displayed decreased expression, while four (*cfa-miR-103*, *cfa-miR-98*, *cfa-let-7b*, and *cfa-let-7c*) showed increased expression, in Stage B1/B2 or C/D compared with Stage A. All 11 of these differed between MMVD Stage A and Stage C/D, while nine differed between Stage A and Stage B1/B2. The expression changes were greater as disease severity increased (Figure 1). Of those 11, six (*cfa-miR-582*, *cfa-miR-487b*, *cfa-miR-103*, *cfa-miR-98*, *cfa-let-7b*, and *cfa-let-7c*) were significantly different between Stages B1/B2 and C/D (Table 1). The expression data of all 277 miRNAs are provided in Table S1.

### 2.2. Potential Role of the Cfa-let-7/cfa-miR-98 Family Members in Canine MMVD

Recent studies in humans have associated the *let-7* family with the development of cardiovascular diseases, and upregulation of *let-7* expression was observed in many patients with cardiovascular diseases, including cardiac hypertrophy, dilated cardiomyopathy, myocardial infarction and CHF [23,24]. Experimental evidence suggests that circulating *let-7b* and cellular *let-7i* might be biomarkers for myocardial infarction and dilated cardiomyopathy, respectively [25,26]. Remarkably, *let-7c* was found to be enriched in cardiac valve in dogs, monkeys and rats [27]. Angiotensin II (*Ang II*) plays an important role in the pathogenesis of CHF secondary to MMVD and other diseases [28]. Recent studies showed that thioredoxin (*Trx1*) negatively regulated *AngII*-induced cardiac hypertrophy by increasing the expression of *let-7*/*miR-98* family members in human CHF and *let-7*/*miR-98* was the downstream effector of *Trx1* [29,30]. In a previous canine gene expression study (National Center for Biotechnology Information’s Gene Expression Omnibus (GEO) Accession No. GSE64544) [31], we found that *Trx1* was upregulated 2.6-fold (*p* = 0.002) in the LV, while angiotensinogen, the precursor of *Ang II*, was downregulated by more than 8-fold (*p* ≤ 0.001) in the mitral valve (MV) of dogs with MMVD. This suggests that the *Trx1-let-7*/*miR-98-Ang II* regulatory circuitry may also exist in canine MMVD and CHF.

Target prediction analysis by TargetScan suggested that tuberous sclerosis 1 (*TSC1*), a gene that encodes hamartin, is a predicted gene target for *cfa-let-7c* (Table S2). Mutations in *TSC1* cause a genetic syndrome called Tuberous sclerosis complex (TSC) [32]. Arrhythmia is relatively common in patients with TSC, and at least 50% of children with TSC developed cardiac rhabdomyomas, which in some cases can lead to CHF [32]. Mutations in *TSC1* have also been associated with mitral valve prolapse [33]. Taken together, our data suggest that *cfa-let-7*/*cfa-miR-98* family may play an important role in MMVD in dogs.

**Table 1:** Heat map of microRNAs differentially expressed between dogs in the three study groups (*n* = 6/group): normal dogs at risk of heart disease (Stage A), asymptomatic dogs with myxomatous mitral valve disease (MMVD) and mild to moderate cardiac enlargement (Stage B1/B2) and dogs with MMVD and congestive heart failure requiring multiple cardiac medications (Stage C/D).

miRNA Name	ANOVA		Stage B1/B2 *vs.* Stage A		Stage C/D *vs.* Stage A		Stage C/D *vs.* Stage B1/B2	
	*p* Value	*FDR* ^a^	*p* Value ^b^	*FC* ^c^	*p* Value	*FC*	*p* Value	*FC*
*cfa-miR-302d*	0.0010	0.0378	0.0050	−2.70	0.0053	−3.51	0.6970	−1.30
*cfa-miR-380*	0.0001	0.0119	0.0020	−2.48	<0.0001	−4.76	0.2020	−1.92
*cfa-miR-874*	0.0016	0.0434	0.0117	−2.37	0.0058	−3.69	0.2547	−1.56
*cfa-miR-582*	<0.0001	0.0004	0.0003	−2.28	<0.0001	−4.71	0.0159	−2.06
*cfa-miR-490*	0.0005	0.0261	0.0101	−2.24	0.0012	−4.03	0.0666	−1.80
*cfa-miR-329b*	0.0019	0.0490	0.0008	−1.96	0.0050	−2.36	0.8497	−1.20
*cfa-miR-487b*	0.0012	0.0427	0.0642	−1.45	0.0025	−3.08	0.0023	−2.12
*cfa-miR-103*	0.0002	0.0140	0.1719	1.29	0.0011	3.81	0.0031	2.96
*cfa-miR-98*	0.0014	0.0428	0.0090	2.15	0.0029	4.07	0.0341	1.90
*cfa-let-7c*	0.0003	0.0218	0.0309	2.28	0.0012	4.67	0.0080	2.05
*cfa-let-7b*	0.0006	0.0269	0.0123	2.38	0.0009	4.03	0.0342	1.69

^a^ False Discovery Rate; ^b^ Student’s *t*-test; ^c^ Fold change: negative numbers indicate decreases in expression, positive numbers for increases in expression. Red, green, and grey colors indicate a significant increase, decrease, and non-significance in expression, respectively.

### 2.3. Cfa-miR-302d as a Potential Negative Regulator of TGF-β Signaling

*TGF-β* signaling pathway has been implicated to play a central role in the pathology of canine MMVD [28,34,35]. In a recent study using human kidney mesangial cells, *miR-302* expression was shown to inhibit TGF-β receptor II (*TβRII)* transcription [36]. As a result, *miR-302d* decreased *TGF-β*-induced epithelial mesenchymal transition and attenuated *TβRII*-mediated production of fibronectin and thrombospondin [36]. The current study showed decreased expression of *cfa-miR-302d* in the serum of dogs with MMVD. This, along with previous observations in dogs with MMVD of increased gene and protein expression of fibronectin and TβRI/TβRII, respectively in the MV [34,35] and increased thrombospondin 1 in LV and MV and thrombospondin 4 in the LV [31], suggest a potential role of *cfa-miR-302d*/*TGF-β* regulatory network in the pathology of MMVD in dogs.

### 2.4. Other MiRNAs

Members of the *miR-103*/*miR-107* family have been implicated in the pathogenesis of cardiovascular disease and were recently shown to regulate insulin sensitivity in obese mice [37], the exact role of *miR-103* in CHF remains unclear. *TβRII* and *TβRIII* are among predicted targets for *cfa-miR-103* (Table S2 ), suggesting a potential role for *cfa-miR-103* in canine MMVD.

*MiR-874* was shown to cause myocardial cell death, an important reason for CHF and myocardial infarction, by targeting the caspase-8 in both *in vitro* and *in vivo* mouse models [38]. Circulating *miR-380* and *miR-582* were reported as a potential noninvasive biomarker for survival prediction after acute myocardial infarction [39] and for detection of deep vein thrombosis [40], respectively. A recent study has demonstrated that *miR-329* is a negative regulator of angiogenesis by directly targeting *CD146* [41]. Gene silencing of the four 14q32 miRNA cluster, including *miR-329* and *miR-487b*, resulted in increased perfusion after ischemia in mice [42]. Additional research is necessary to unravel potential roles of these miRNAs in canine MMVD.

Neither *cfa-miR-133* nor *cfa-miR-30* reached statistical significance in our ANOVA analysis. Although this differs from the findings of Hulanicka *et al.* [20], the group did not report ANOVA analysis, instead employed a series of *t*-tests to address a multisample hypothesis [43]. Chen *et al.* also failed to observe changes in *cfa-miR-133* expression in LV cardiomyocyte of an induced model of canine CHF [21].

Besides *let-7c*, both *miR-125b* and *miR-204* were enriched in cardiac valves of rat, dog and monkey [27]. Inhibitory interaction between *miR-204* and *TβRII* was identified *in silico* and demonstrated experimentally in human tissue culture cells [27]. Although neither showed significance in our overall ANOVA analysis (*FDR* = 0.12 and *FDR* = 0.43, respectively), simple *t*-tests suggested they were different between stages B1/B2 and C/D (*p* = 0.03 and *p* = 0.01, respectively). This suggests that the lack of statistical significance may reflect a type 2 error (false negative) due to small sample size.

### 2.5. Limitations

This pilot study was limited by a small sample size and heterogeneity of the dogs from which serum samples were collected. Future investigations with larger cohorts of animals are required before any clinical applications can be considered. It was also not possible to determine the effects of medications on expression due to the small number of animals and variable medications they were receiving. This warrants additional research in future studies. In addition, *in situ* hybridization can provide additional confirmation on some of the important miRNAs. Nonetheless, the findings reported here suggest there is an opportunity for using some of these circulating miRNAs as biomarkers for diagnosis, prognosis or monitoring response to treatment in MMVD in dogs. Our pilot study will provide a platform and knowledge for future studies.

## 3. Experimental Section

### 3.1. Animals and Sample Collection

For the current study, 18 dogs of various breeds were classified either as being healthy or with MMVD by echocardiography performed or evaluated by a board-certified veterinary cardiologist, pathological examination of the heart, or both (Table S3). Dogs were further classified into one of three groups of six dogs each using the American College of Veterinary Internal Medicine guidelines for diagnosis of MMVD [4]: normal dogs at risk of heart disease (Stage A: 2 neutered males [MN], 4 spayed females [FS]; age 8.5 ± 2.7 years; body weight 10.2 ± 13.6 kg), asymptomatic dogs with MMVD and mild to moderate cardiac enlargement (Stage B1/B2: 2 MN, 4 FS; age 10.9 ± 2.7 years; body weight 13.5 ± 8.8 kg) and dogs with MMVD and CHF requiring multiple cardiac medications (Stage C/D: 2 MN, 4 FS; age 10.3 ± 3.4 years; body weight 10.4 ± 6.3 kg). Venous blood was collected and serum was separated and frozen at −80 °C until analysis. The study protocol was reviewed and approved by Nestlé Purina’s Institutional Animal Care and Use Committee.

### 3.2. Quantitative RT-PCR, Data Normalization and Statistical Analysis

Serum sample processing and real-time PCR assay were performed at a commercial laboratory using the miScript miRNA PCR Array system (Qiagen, Fredrick, MD, USA) [44]. Total RNA was extracted and purified from 200 microliters serum samples using the miRNeasy Serum/Plasma Kit from Qiagen. The cycle threshold (*C*_t_) value was measured for each miRNA. The normalization factor for each sample was determined according to Mestdagh *et al.* [45]. *C*_t_ values that were undetermined or greater than 30 were reassigned with 30. Normalized *C*_t_ (Δ*C*_t_) was obtained by subtracting the normalization factor from each *C*_t_ value. The expression of each miRNA is reported as 2^−∆*C*t^. ANOVA was performed to identify differentially expressed miRNAs among the three stages of MMVD. *FDR* was calculated to control multiple testing errors [46]. The miRNAs with *FDR* < 0.05 were deemed as significant. Significant miRNAs were subject to Student’s *t*-test with equal variance to compare the pairwise difference between stages. Expression fold change also was calculated.

### 3.3. Computational Prediction of MiRNA Targets

MiRNA targets were predicted using the software TargetScan [47]. Briefly, the software scans for the presence of conserved 7 or 8 mer that matches the miRNA’s seed region, which is the nucleotide sequence in positions 2–7 of a mature miRNA. Predicted targets of *cfa-let-7b*, *cfa-let-7c*, and *cfa-miR-103* were downloaded from the TargetScan website.

## 4. Conclusions

The study surveyed the expression profiling of 277 circulating miRNAs in the serum of dogs at different stages of MMVD and CHF using quantitative real-time PCR array system. Eleven miRNAs were differentially expressed (*FDR* < 0.05). Dogs in Stage B1/B2 or C/D had four upregulated miRNAs, including three *cfa-let-7*/*cfa-miR-98* family members, while seven others were downregulated, compared to Stage A. The expression changes were greater as disease severity increased. Our study suggests that there is an opportunity for using some of these circulating miRNAs as biomarkers for diagnosis, prognosis or monitoring response to treatment in MMVD in dogs. Further investigation of these miRNAs may also shed light on genetic regulatory pathways on canine MMVD.

Dogs and humans share some similarities in MMVD, including degenerative valvular structure, expression patterns of extracellular matrix proteins, and some common signaling pathways [48]. Therefore, results from this study, including changes in the *cfa-let-7*/*cfa-miR-98* family members, may be relevant to the study of human MMVD.

## Supplementary Materials

Supplementary materials can be found at http://www.mdpi.com/1422-0067/16/06/14098/s1.
